# Exploring disparities in satisfaction with obstetric-gynecological care among insured and uninsured women in Almaty, Kazakhstan: a comparative cross-sectional study

**DOI:** 10.3389/fgwh.2025.1580888

**Published:** 2025-07-25

**Authors:** Sholpan Aliyeva, Vyacheslav Lokshin, Maksut Kamaliev, Sholpan Sarmuldayeva, Gani Kaldybayev, Oxana Tsigengagel

**Affiliations:** ^1^ Department of Clinical Disciplines, Kazakhstan’s Medical University “Kazakhstan School of Public Health”, Almaty, Kazakhstan; ^2^ Department of Gynecological, JSC Central Clinical Hospital, Almaty, Kazakhstan; ^3^ Department of Assisted Reproductive Technologies, International Clinical Centre of Reproduction “PERSONA”, Almaty, Kazakhstan; ^4^ Department of Health Management, Kazakhstan’s Medical University “Kazakhstan School of Public Health”, Almaty, Kazakhstan; ^5^ Department of Medicine, Al-Farabi Kazakh National University, Almaty, Kazakhstan; ^6^ Birth Center, Karasay Clinical Multidisciplinary Central District Hospital, Kaskelen, Kazakhstan; ^7^ Department of Biostatistics, Bioinformatics, and Information Technologies, Astana Medical University, Astana, Kazakhstan

**Keywords:** health insurance, patient satisfaction, socioeconomic factors, outpatients, health equity, healthcare disparities, obstetrics and gynecology, Kazakhstan

## Abstract

**Background:**

Patient satisfaction is a key indicator of healthcare quality. Although crucial, limited research has explored factors contributing to satisfaction disparities in outpatient obstetric-gynecological care, particularly in Kazakhstan. The objective of the study is to explore disparities in satisfaction with obstetric-gynecological care between insured and uninsured women in Almaty, Kazakhstan, and to identify the key determinants of patient satisfaction.

**Methods:**

A cross-sectional survey was conducted among 107 insured and uninsured patients over three months in early 2024 at a hospital in the Almaty region, Kazakhstan. Using a five-point Likert scale, a structured questionnaire assessed socio-demographics, patient-reported experiences with the care process, and overall satisfaction across 16 dimensions. The survey instrument was pilot-tested and demonstrated strong internal consistency (Cronbach's alpha = 0.83). Chi-square tests examined associations, and multivariable logistic regression identified key predictors of patient satisfaction.

**Results:**

A multivariable analysis revealed a “satisfaction paradox”: insured patients had lower odds of being satisfied compared to uninsured patients (AOR = 0.15, 95% CI: 0.03–0.81). Specifically, a lack of insurance was associated with higher reported satisfaction in doctor-patient communication (OR = 1.8) and nursing care (OR = 2.1). Other significant predictors of satisfaction included having kidney disease and a shorter hospital stay.

**Conclusions:**

Our findings suggest that expanding insurance coverage is necessary for access but insufficient for ensuring patient satisfaction. The observed “satisfaction paradox” highlights that policy must adopt a dual focus: promoting enrolment while simultaneously improving the quality of patient-provider interactions to meet the higher expectations of insured patients.

## 1 Introduction

Kazakhstan's healthcare system has recently undergone a fundamental transformation, moving from a fully state-funded model to a mixed system based on Mandatory Social Health Insurance (MSHI) (1, 2). This reform, launched in 2020, created a two-tiered system: a basic **“**Guaranteed Volume of Free Medical Care” (GVFMC) available to all citizens, and an expanded MSHI package available only to insured individuals. By its very design, this structure creates systemic disparities in access to care, a phenomenon supported by international evidence showing that uninsured populations consistently face barriers to receiving timely, high-quality care (3). These disparities are particularly acute in vital areas such as obstetrics and gynaecology (OB/GYN), where the quality and timeliness of care directly impact women's health and pregnancy outcomes (4). Few studies specifically focus on the issue of insured vs. uninsured women in obstetrics and gynecology, and even fewer examine healthcare access and satisfaction in other countries (5).

The importance of patient satisfaction in evaluating healthcare systems cannot be overstated. Beyond being a measure of comfort, it serves as a crucial proxy for healthcare quality, directly correlating with improved clinical outcomes and trust in medical institutions (6). A significant determinant of this satisfaction is the burden of out-of-pocket expenditure. For uninsured individuals, who are entitled only to the basic GVFMC package, the necessity of paying for additional services creates a direct financial barrier that not only limits their access to comprehensive care but also negatively shapes their overall healthcare experience and their evaluation of its quality (7).

Patient satisfaction with insurance status has been the subject of research in various nations. For instance, Community-Based Health Insurance (CBHI) and other comprehensive financial risk protection programs have been introduced in Ethiopia, reaching a sizable section of the populace, particularly those employed in the unorganized sector. Research shows that 54% and 93.38% of patients are satisfied with their healthcare treatments. Factors such as long wait times, availability of medications, and the friendliness of healthcare staff significantly influence this satisfaction. These findings underscore the importance of financial risk protection in shaping healthcare satisfaction and access, which is crucial for achieving the global commitment to Universal Health Coverage (UHC) by 2030. However, despite these initiatives, disparities in healthcare access and satisfaction persist, and there is a limited amount of research focusing on the differences between insured and uninsured women regarding obstetric-gynecological care (8–11).

Despite the global recognition of this issue, there is a significant research gap concerning the impact of Kazakhstan's new MSHI system on patient satisfaction. Specifically, there is a lack of research on the differences in experience between insured and uninsured women seeking obstetric and gynaecological care. This field is an ideal case for analysis as it encompasses a wide spectrum of services (from emergency care covered by GVFMC to elective procedures available only through MSHI), serves a universal need, and directly affects national demographic and health indicators.

Therefore, the primary objective of this study is to analyze the disparities in patient satisfaction with obstetric-gynecological care between insured and uninsured women in Almaty, Kazakhstan. We specifically aim to determine the effect of health insurance status on patient satisfaction while controlling for key socio-demographic and systemic factors.

## 2 Materials and methods

### 2.1 Study setting, period, and design

This study used a cross-sectional methodology to examine the factors associated with patient satisfaction in obstetric-gynecological care in a hospital in the Almaty, Kazakhstan. This hospital was chosen because it is one of the largest public multidisciplinary facilities in the Almaty region, serving a diverse urban and suburban population, which ensures a sufficient and representative sample of both insured and uninsured patients seeking obstetric-gynecological care. There were 107 participants, both with and without insurance. Data collection spanned three months, from January to March 2024, utilizing structured questionnaires and hospital documentation.

### 2.2 Participants

The study population comprised adult patients who visited the outpatient obstetric-gynecological department of the selected hospital during the study period. Inclusion Criteria: (1) Adult patients (aged 18 years and older) capable of providing informed consent. (2) Patients seeking obstetric-gynecological care during the data collection period. Exclusion Criteria: (1) Critically ill patients. This criterion was applied to ensure that all participants could provide fully informed consent and to prevent their satisfaction ratings from being disproportionately influenced by the severity of their condition rather than the standard service quality. (2) Patients who were unable or unwilling to provide written informed consent to participate in the study.

### 2.3 Sample size determination

The sample size for this study was determined using an online Raosoft calculator, aiming to compare two independent proportions. Based on the findings of a previous comparative study on patient satisfaction, which reported satisfaction rates of 68.8% for insured and 62.4% for uninsured patients, the calculation was performed (12). Setting the confidence interval at 95% and the statistical power at 80%, the recommended sample size was calculated. After accounting for a potential non-response rate, a target sample was established. The final sample consisted of 107 participants, which represents the number of eligible and consenting patients successfully recruited during the study period. We acknowledge that this sample size is relatively small, which means the study is exploratory and was not sufficiently powered to draw definitive conclusions, particularly for the multivariable analysis. This is addressed as a key limitation of the study.

### 2.4 Variables and measurements

A structured questionnaire adapted from similar studies was developed for this research (13–16). The final instrument consists of three sections. The first section collects demographic information, including age, marital status, education level, residence, income, and insurance status. The second section explores factors related to hospital services, hospitalization methods, and patient involvement in treatment decisions for insured and uninsured individuals. The final section evaluates patient satisfaction through 16 statements, with responses measured on a five-point scale from 1 (strongly disagree) to 5 (strongly agree).

To ensure the validity and reliability of the questionnaire, a rigorous process was followed. First, to ensure linguistic accuracy, the English version was translated into Kazakh and then back-translated into English by independent language experts. Second, the Kazakh version underwent a pilot study at a nearby health center to assess its clarity and comprehensibility, after which minor wording adjustments were made based on feedback. The internal consistency of the 16-item satisfaction scale was confirmed with a Cronbach's alpha coefficient of 0.83, indicating strong reliability. This was initially assessed on a pilot sample before main data collection and subsequently verified on the final dataset.

For analysis, a total satisfaction score was calculated for each participant by summing the responses from the 16 items. For analysis, this score was then dichotomized. Because the distribution of the satisfaction scores was skewed, the sample median was used as the cut-off point, as it is a more robust measure of central tendency than the mean. Specifically, the data exhibited a negative skew, indicating a higher concentration of responses towards the upper end of the satisfaction scale. Participants with scores at or above the median were classified as “satisfied,” and those with scores below the median were classified as “not satisfied”. The full questionnaire is available as Supplementary File S1.

### 2.5 Data collection and procedures

Data were collected remotely from January to March 2024. The recruitment and data collection process followed a structured protocol to ensure participant anonymity and voluntary participation. Participants were recruited through a database of patients from the participating hospital who had previously given explicit consent to be contacted for future research purposes. Individual invitations were sent to potential participants via email or WhatsApp. These invitations provided a brief overview of the study's purpose and a secure link to the online questionnaire (17, 18).

The first page of the online survey (hosted on Google Forms) served as an information and consent form. It detailed the study's objectives, the voluntary nature of participation, and the measures taken to ensure data confidentiality. Participants were required to provide explicit consent by checking a box stating, “*I have read the information above and I voluntarily agree to participate in this study*” before they could proceed to the questionnaire.

The questionnaire was self-administered online. All data were collected anonymously. After the data collection period, aggregate, non-identifiable data points were cross-referenced with general hospital statistics by authorized personnel to check for consistency, while strictly maintaining patient privacy.

### 2.6 Statistical analysis

The data were analyzed using SPSS software version 27.0 (IBM Corp., Armonk, NY, USA). Descriptive statistics, including frequencies, proportions, and means, were calculated, and the results were presented in text and tables where appropriate. For bivariate analysis, variables with a *p*-value of less than 0,05 (5%) were selected for further evaluation using multivariable logistic regression. A multivariable logistic regression model was developed to identify key predictors of patient satisfaction. The outcome variable was the dichotomized satisfaction score (“satisfied” vs. “not satisfied”). Independent variables with a *p*-value < 0.25 in the bivariate analysis were included in the multivariable model. Before applying the model, tests for goodness-of-fit, overall classification, and Pseudo R² were conducted. Adjusted odds ratios (AOR) with 95% confidence intervals (CI) were reported to assess the strength of associations, and a *p*-value of <0.05 was considered statistically significant for all tests.

## 3 Results

Data for all variables included in the analysis were complete for all 107 participants.

### 3.1 Socio-Demographic characteristics of the study group

The study included 107 female patients. The socio-demographic characteristics of the overall sample, stratified by insurance status, are presented in Table 1. A bivariate analysis using chi-square tests revealed that the insured and uninsured groups differed significantly by marital status (*p* = 0.030), a presented in Table 1. A bivariate analysis comparing the insured and uninsured groups revealed a statistically significant difference in marital status (*p* = 0.030), with insured patients more likely to be married. No other significant differences were observed for key demographic variables such as age, education, or income level.

**Table 1:** Socio-demographic characteristics of insured and uninsured patients in a hospital, Almaty region, Kazakhstan (*n* = 107).

Variables	Categories	Insured (%)	Uninsured (%)	Chi-square (*χ*²)	(*p*-value)
Age	<25 years	38 (44,2%)	14 (66,7%)	6.03	0.116
	25–34 years	20 (23,3%)	5 (23,8%)		
	35–44 years	16 (18,6%)	0 (0,0%)		
	45–54 years	12 (14,0%)	2 (9,5%)		
Marital status	Married	58 (67.4%)	7 (33.3%)	8.91	0.030*
	Single	13 (15.1%)	7 (33.3%)		
	Divorced	14 (16.3%)	7 (33.3%)		
	Widowed	1 (1.2%)	0 (0.0%)		
Educations	Secondary	40 (46,5%)	11 (52,4%)	0.40	0.818
	Higher	39 (45,3%)	9 (42,9%)		
	Postgraduate	7 (8,1%)	1 (4,8%)		
Residence	Urban	59 (68,6%)	27 (31,4%)	0.00	1.000
	Rural	15 (71,4%)	6 (28,6%)		
Income	<129,277 tenge	62 (72,1%)	24 (27,9%)	2.17	0.140
	>129,277 tenge	19 (90,5%)	2 (9,5%)		

Note: *p*-values are based on Pearson's chi-square test. For variables with expected frequencies <5, Fisher's exact test was applied. For marital status, both tests yielded consistent results: χ² = 8.91, *p* = 0.030 (chi-square); *p* ≈ 0.049 (Fisher's exact).

*A *p*-value < 0.05 was considered statistically significant.

### 3.2 Patient satisfaction, and healthcare utilization by insurance status

As shown in Table 2, uninsured patients generally reported higher levels of satisfaction across multiple service domains compared to insured patients. For example, 95.2% of uninsured patients were fully satisfied with the hospitalization process, compared to 90.7% of insured patients.

**Table 2:** Comparative analysis of patient satisfaction with hospital services based on insurance Status in the Almaty region, Kazakhstan (*n* = 107) .

Variables	Insurance Status	Categories Satisfied	Somewhat Satisfied	Somewhat Dissatisfied	Dissatisfied
		*N* (%)	*N* (%)	*N* (%)	*N* (%)
Perception of the Hospitalization Process	Uninsured	20 (95,2%)	0 (0,0%)	0 (0,0%)	1 (4,8%)
	Insured	78 (90,7%)	1 (1,2%)	3 (3,5%)	3 (3,5%)
Perception of Hospitalization Duration	Uninsured	16 (76,2%)	0 (0,0%)	0 (0,0%)	5 (23,8%)
	Insured	54 (62,8%)	2 (2,3%)	2 (2,3%)	28 (32,6%)
Perception of Involvement in Treatment and/or Delivery Decisions	Uninsured	19 (90,5%)	0 (0,0%)	0 (0,0%)	2 (9,5%)
	Insured	61 (70,9%)	1 (1,2%)	2 (2,3%)	22 (25,6%)
Doctors' Attitude, Behavior, and Communication	Uninsured	16 (76,2%)	0 (0,0%)	0 (0,0%)	5 (23,8%)
	Insured	65 (75,6%)	3 (3,5%)	1 (1,2%)	17 (19,8%)
Nurses' Attitude, Behavior, and Communication	Uninsured	21 (100,0%)	0 (0,0%)	0 (0,0%)	0 (0,0%)
	Insured	83 (96,5%)	1 (1,2%)	2 (2,3%)	0 (0,0%)
Other Clinic Staff's Attitude, Behavior, and Communication	Uninsured	21 (100,0%)	0 (0,0%)	0 (0,0%)	0 (0,0%)
	Insured	82 (95,3%)	4 (4,7%)	0 (0,0%)	0 (0,0%)
Waiting Time for Hospitalization or Assistance	Uninsured	21 (100,0%)	0 (0,0%)	0 (0,0%)	0 (0,0%)
	Insured	81 (94,2%)	5 (5,8%)	0 (0,0%)	0 (0,0%)
Quality of Room Accommodation	Uninsured	21 (100,0%)	0 (0,0%)	0 (0,0%)	0 (0,0%)
	Insured	82 (95,3%)	3 (3,5%)	1 (1,2%)	0 (0,0%)
Availability, Quality, and Cleanliness of Toilets	Uninsured	21 (100,0%)	0 (0,0%)	0 (0,0%)	0 (0,0%)
	Insured	82 (95,3%)	2 (2,3%)	2 (2,3%)	0 (0,0%)
Availability of Hot Water, Ability to Take a Shower	Uninsured	21 (100,0%)	0 (0,0%)	0 (0,0%)	0 (0,0%)
	Insured	83 (96,5%)	2 (2,3%)	1 (1,2%)	0 (0,0%)
Quality of Provided Food	Uninsured	21 (100,0%)	0 (0,0%)	0 (0,0%)	0 (0,0%)
	Insured	79 (91,9%)	5 (5,8%)	2 (2,3%)	0 (0,0%)
Quality of Diagnostic Procedures	Uninsured	20 (95,2%)	1 (4,8%)	0 (0,0%)	0 (0,0%)
	Insured	82 (95,3%)	3 (3,5%)	1 (1,2%)	0 (0,0%)
Quality of Procedures and Interventions	Uninsured	21 (100,0%)	0 (0,0%)	0 (0,0%)	0 (0,0%)
	Insured	82 (95,3%)	3 (3,5%)	3 (3,5%)	0 (0,0%)
Medication Supply	Uninsured	81 (94,2%)	3 (3,5%)	2 (2,3%)	0 (0,0%)
	Insured	81 (94,2%)	3 (3,5%)	2 (2,3%)	0 (0,0%)
Overall Medical Service	Uninsured	21 (100,0%)	21 (100,0%)	21 (100,0%)	0 (0,0%)
	Insured	82 (95,3%)	4 (4,7%)	0 (0,0%)	0 (0,0%)

Table 3 shows a significant difference in the pathway to care (*p* = 0.010), with a higher proportion of insured patients utilizing emergency services. A chi-square test was performed to examine the relationship between insurance status and the final dichotomized satisfaction outcome, confirming a significant relationship (*χ*² = 8.91, *p* = 0.030).

**Table 3:** Comparison of hospitalization methods between insured and uninsured patients: distribution and statistical analysis.

Variables	Categories	Insured (%)	Uninsured (%)	*P*-value
Hospitalization Method	Emergency Care	54 (79,4%)	14 (20,6%)	0,010
	Self-Referral	24 (96,0%)	1 (4,0%)	
	Referral by Doctor	8 (57,1%)	6 (42,9%)	

### 3.3 Multivariable analysis of factors associated with patient satisfaction

The results of the multivariable logistic regression for patient satisfaction are presented in Table 4. Several factors were significantly associated with lower odds of patient satisfaction. Insured patients had 85% lower odds of being satisfied compared to uninsured patients [Adjusted Odds Ratio (AOR) = 0.15; 95% CI: 0.03–0.81; *p* = 0.03], patients with kidney disease had 87% lower odds of being satisfied (AOR = 0.13; 95% CI: 0.03–0.63; *p* = 0.01), and patients with shorter hospital stays had 38% lower odds of being satisfied (AOR = 0.62; 95% CI: 0.41–0.93; *p* = 0.02). In contrast, only one factor was associated with higher odds of satisfaction: experiencing complications related to the primary diagnosis had 77% higher odds of being satisfied (AOR = 1.77; 95% CI: 1.00–3.34; *p* = 0.05). Other demographic and clinical factors were not significantly associated with patient satisfaction in the final model (*p* > 0.05).

**Table 4:** Binary and multivariable logistic regression analysis to identify factors associated with satisfaction of patients (*n* = 107).

Variables	Not satisfied	Satisfied	At binary level		Multi-variable level	
			COR (95% CI)	*p*-value	AOR (95% CI)	*p*-value
Age	50	57	0.95 (0.90, 1.01)	0.15	0.96 (0.91, 1.01)	0.13
Gender	45	63	2.13 (0.98, 4.64)	0.057	1.91 (0.88, 4.17)	0.10
Region	55	52	0.45 (0.10, 2.17)	0.32	0.36 (0.08, 1.71)	0.20
Education	60	47	0.51 (0.16, 1.67)	0.26	0.55 (0.17, 1.70)	0.30
Income	40	67	0.39 (0.07, 2.24)	0.29	0.33 (0.05, 1.85)	0.21
Insurance Status	30	77	0.11 (0.02, 0.67)	0.017	0.15 (0.03, 0.81)	0.03
Kidney Disease	20	87	0.16 (0.03, 0.83)	0.03	0.13 (0.03, 0.63)	0.01
Vascular Disease	25	82	0.06 (0.002, 2.19)	0.13	0.05 (0.002, 1.74)	0.10
Days of Hospitalization	35	72	0.64 (0.42, 0.98)	0.04	0.62 (0.41, 0.93)	0.02
Complications (Main Dx)	15	92	1.82 (0.94, 3.48)	0.07	1.77 (1.00, 3.34)	0.05

## 4 Discussion

This study examined the factors influencing patient satisfaction among women receiving OB/GYN care in Almaty, Kazakhstan. Our analysis revealed a key paradoxical finding: uninsured patients reported higher satisfaction in several key domains of care, despite having poorer access to healthcare services.

We hypothesize this finding is linked to household-level decision-making. Insurance participation is heavily influenced by household structure, and some families may consciously forgo commercial insurance due to a lack of trust or a perceived lack of value (19–21). Consequently, their interaction with the healthcare provider becomes a straightforward service transaction, free from the bureaucratic layers that often diminish the experience for insured patients.

An analysis of patient satisfaction revealed that uninsured patients reported higher satisfaction levels in most aspects of care, including the hospitalization process (95.2%) and involvement in treatment decisions (90.5%). This “satisfaction paradox” is a recognized phenomenon in health services research, often explained by an “expectations-experience gap,” where lower baseline expectations among underserved populations lead to higher reported satisfaction for any given level of care (22, 23). It is crucial to interpret this finding correctly: it does not suggest that a lack of insurance is beneficial or that enrolment efforts should be diminished. Rather, it highlights a critical system-level challenge. While insurance is essential for ensuring financial protection and improving access to care, as our utilization data confirms, the system must also be prepared to meet the higher expectations that come with coverage.

In contrast, insured patients expressed slightly greater dissatisfaction regarding doctor communication and involvement in treatment decisions. This dissatisfaction may stem from their unmet expectations for more frequent and comprehensive healthcare access. Improving patient-provider communication and promoting shared decision-making could empower patients to engage more actively in their healthcare choices (24). A key positive finding was the high satisfaction with nursing care, with 100% of uninsured patients and 96.5% of insured patients expressing satisfaction. This finding underscores the pivotal role of nursing care in shaping the overall patient experience.

An interesting aspect of our findings is the lack of a significant association between most socio-demographic factors—such as age, education and income—and patient satisfaction in the multivariate model. This contrasts with some studies that suggest that demographic characteristics, such as older age or marital status, are often associated with higher levels of satisfaction (25). However, our result is consistent with other studies claiming that the influence of demography may be inconsistent or attenuated when clinical and systemic factors are taken into account (26, 27). This suggests that in the context of Kazakhstan's health care system, a patient's specific health status and their direct interaction with the care delivery process may be more powerful factors shaping their experience than their demographic profile.

Instead, our model revealed that clinical and systemic factors were strong predictors of satisfaction, sometimes in counterintuitive ways. For instance, patients with kidney disease reported significantly lower satisfaction, likely reflecting the heavy burden of managing a chronic condition. More surprisingly, patients who experienced complications reported higher satisfaction. This may be explained by the increased attention and more frequent interactions with healthcare providers that often accompany complications. This aligns with studies emphasizing that a strong doctor-patient bond and compassionate communication are key to patient satisfaction, suggesting that increased contact time, even if necessitated by adverse events, can paradoxically strengthen the patient-provider relationship (28–30). Future research with a larger, adequately powered sample should also consider conducting stratified analyses to explore whether the determinants of patient satisfaction differ between insured and uninsured populations, as this could reveal unique patterns within each group.

## 5 Limitations of the study

This study has several limitations. This study has several important limitations that frame its findings as exploratory. The primary limitation is the small sample size (*n* = 107), which was not sufficient to achieve conventional levels of statistical power. An *a priori* power calculation based on the effects reported by Shure et al. (2023) indicates that a sample of approximately 1,720 participants would be required to achieve 80% power; thus, our study was significantly underpowered. Consequently, the results, particularly from the multivariable regression, should be interpreted with caution as they may lack stability and require confirmation in larger studies. Other limitations include the cross-sectional design, which prevents the establishment of causal relationships, and the study's single-center nature, which may limit generalisability. Finally, the dichotomization of the satisfaction score, while done to handle a skewed distribution, can reduce statistical power and is another key limitation.

## 6 Conclusion

This study's central finding is that expanding insurance coverage is a necessary, but insufficient, condition for achieving true healthcare equity in Kazakhstan. While insured patients had better access to services, they paradoxically reported lower satisfaction in key domains. This “satisfaction paradox” suggests the patient experience is shaped as much by subjective expectations as it is by objective barriers like finances. Consequently, policy must adopt a dual focus: continuing to remove financial barriers for vulnerable populations while simultaneously investing in the quality of systemic interactions, particularly communication and shared decision-making. Future research should therefore explore interventions targeting these patient expectations to ensure that improved access consistently translates into a high-quality care experience for all.

## Data Availability

The raw data supporting the conclusions of this article will be made available by the authors, without undue reservation.

## References

[1] Rechel B Sydykova A Moldoisaeva S Sodiqova D Spatayev Y Ahmedov M Primary care reforms in Central Asia—on the path to universal health coverage? Health Policy Open. (2023) 5:100110. 10.1016/j.hpopen.2023.100110 38073710 PMC10704368

[2] Aliyeva S Lokshin V Kamaliev M Sarmuldayeva S Tsigengagel O. Health professionals’ perspectives on challenges in providing obstetrics and gynecology care in Kazakhstan: a qualitative study. Int J Healthc Manag. (2024):1–7. 10.1080/20479700.2024.2323835

[3] Sommers BD Gawande AA Baicker K. Health insurance coverage and health — what the recent evidence tells us. N Engl J Med. (2017) 377:586–93. 10.1056/nejmsb1706645 28636831

[4] Aliyeva SU Atabayeva AK Ansatbayeva TN Kokayeva EA Sagalbayeva UY Tsigengagel OP. Key predictors of satisfaction among gynecological patients in Almaty, Kazakhstan: a multivariate analysis. Reprod Med. (2025) 1:49–56. 10.37800/RM.1.2025.472

[5] Aragon SJ Gesell SB. A patient satisfaction theory and its robustness across gender in emergency departments: a multigroup structural equation modeling investigation. Am J Med Qual. (2003) 18:229–41. 10.1177/106286060301800603 14717381

[6] Doyle C Lennox L Bell D. A systematic review of evidence on the links between patient experience and clinical safety and effectiveness. BMJ Open. (2013) 3:e001570. 10.1136/bmjopen-2012-001570 23293244 PMC3549241

[7] WHO Regional Office for Europe. Health Financing Progress Matrix Assessment: Kazakhstan 2023: Summary of Findings and Recommendations. Copenhagen: World Health Organization, Regional Office for Europe (2025). Available online at: https://www.who.int/europe/publications/i/item/WHO-EURO-2025-11483-51255-78136 (Accessed July 19, 2025).

[8] Alemayehu YK Dessie E Medhin G Birhanu N Hotchkiss DR Teklu AM The impact of community-based health insurance on health service utilization and financial risk protection in Ethiopia. BMC Health Serv Res. (2023) 23:67. 10.1186/s12913-022-09019-6 36683041 PMC9869550

[9] Fetene SM Mengistu MY Aschalew AY. Effectiveness and impact of community-based health insurance on health service utilization in northwest Ethiopia: a quasi-experimental evaluation. Front Public Health. (2023) 11:1078462. 10.3389/fpubh.2023.1078462 38026288 PMC10679351

[10] Bayked EM Toleha HN Kebede SZ Workneh BD Kahissay MH. The impact of community-based health insurance on universal health coverage in Ethiopia: a systematic review and meta-analysis. Glob Health Action. (2023) 16:2189764. 10.1080/16549716.2023.2189764 36947450 PMC10035959

[11] Getaneh MM Bayked EM Workneh BD Kahissay MH. Satisfaction of beneficiaries with community-based health insurance and associated factors in Legambo district, North-East Ethiopia: a cross-sectional study. Front Public Health. (2023) 11:1127755. 10.3389/fpubh.2023.1127755 37261241 PMC10227519

[12] Shure G Gamachu M Mitiku H Deressa A Eyeberu A Mohammed F Patient satisfaction and associated factors among insured and uninsured patients in Deder general hospital, eastern Ethiopia: a facility-based comparative cross-sectional study. Front Med (Lausanne). (2023) 10:1259840. 10.3389/fmed.2023.1259840 38204483 PMC10777387

[13] Geberu DM Biks GA Gebremedhin T Mekonnen TH. Factors of patient satisfaction in adult outpatient departments of private wing and regular services in public hospitals of Addis Ababa, Ethiopia: a comparative cross-sectional study. BMC Health Serv Res. (2019) 19:869. 10.1186/s12913-019-4685-x 31752821 PMC6873435

[14] Eshetie G Feleke A Genetu M. Patient satisfaction and associated factors among outpatient health service users at primary hospitals of North Gondar, Northwest Ethiopia, 2016. Adv Public Health. (2020) 2020:6102938. 10.1155/2020/6102938

[15] Mekonen AM Gebregziabher MG Teferra AS. The effect of community based health insurance on catastrophic health expenditure in Northeast Ethiopia: a cross sectional study. PLoS One. (2018) 13:e0205972. 10.1371/journal.pone.0205972 30335838 PMC6193712

[16] Fenny Ao Enemark U Asante FA Hansen KS. Patient satisfaction with primary health care—a comparison between the insured and non-insured under the national health insurance policy in Ghana. Glob J Health Sci. (2014) 6:9. 10.5539/gjhs.v6n4p9 24999137 PMC4825362

[17] Regmi PR Waithaka E Paudyal A Simkhada P Van Teijlingen E. Guide to the design and application of online questionnaire surveys. Nepal J Epidemiol. (2017) 6:640. 10.3126/nje.v6i4.17258 PMC5506389 28804676

[18] Hulley SB Cummings SR Browner WS Grady D Newman TB. Designing cross-sectional and cohort studies. In: Hulley SB Cummings SR Browner WS Grady D Newman TB, editors. Designing Clinical Research. 4th ed. Philadelphia: Lippincott Williams & Wilkins (2013). p. 85–96.

[19] Xiao W. Effects of marital status on household commercial health insurance participation behavior. J Interdiscip Math. (2018) 21:397–407. 10.1080/09720502.2017.1420569

[20] Sohn H. Health insurance and risk of divorce: does having your own insurance matter? J Marriage Fam. (2015) 77:982–95. 10.1111/jomf.12195 26949269 PMC4772968

[21] Dror DM Shahed Hossain SA Majumdar A Koehlmoos TLP John D Panda PK. What factors affect voluntary uptake of community-based health insurance schemes in low- and middle-income countries? A systematic review and meta-analysis. PLoS One. (2016) 11:e0160479. 10.1371/journal.pone.0160479 27579731 PMC5006971

[22] Ferreira DC Vieira I Pedro MI Caldas P Varela M. Patient satisfaction with healthcare services and the techniques used for its assessment: a systematic literature review and a bibliometric analysis. Healthcare. (2023) 11:639. 10.3390/healthcare11050639 36900644 PMC10001171

[23] Prang KH Canaway R Bismark M Dunt D Kelaher M. Associations between patient experiences and clinical outcomes: a cross-sectional data linkage study of the Australian private healthcare sector. BMJ Open Qual. (2019) 8:e000637. 10.1136/bmjoq-2019-000637 31523739 PMC6711428

[24] Kebede KM Geberetsadik SM. Household satisfaction with community-based health insurance scheme and associated factors in piloted Sheko district; Southwest Ethiopia. PLoS One. (2019) 14:e0216411. 10.1371/journal.pone.0216411 31083706 PMC6513074

[25] Adhikari M Paudel NR Mishra SR Shrestha A Upadhyaya DP. Patient satisfaction and its socio-demographic correlates in a tertiary public hospital in Nepal: a cross-sectional study. BMC Health Serv Res. (2021) 21:135. 10.1186/s12913-021-06155-3 33579283 PMC7881603

[26] Birkeland S Bismark M Barry MJ Möller S. Personality characteristics associated with satisfaction with healthcare and the wish to complain. BMC Health Serv Res. (2022) 22:1305. 10.1186/s12913-022-08688-7 36320078 PMC9628068

[27] Fang J Liu L Fang P. What is the most important factor affecting patient satisfaction—a study based on gamma coefficient. Patient Prefer Adherence. (2019) 13:497–513. 10.2147/PPA.S197015 31114168 PMC6489650

[28] Moslehpour M Shalehah A Rahman FF Lin KH. The effect of physician communication on inpatient satisfaction. Healthcare (Switzerland). (2022) 10:463. 10.3390/healthcare10030463 PMC8954154 35326941

[29] Ha JF Longnecker N. Doctor-patient communication: a review. Ochsner Journal. (2010) 10:84–8. 10.3329/jbcps.v32i2.26036 PMC3096184 21603354

[30] Zhang X Li L Zhang Q Le LH Wu Y. Physician empathy in doctor-patient communication: a systematic review. Health Commun. (2024) 39:1027–37. 10.1080/10410236.2023.2201735 37062918

