# Supplementary material for: Exploring disparities in satisfaction with obstetric-gynecological care among insured and uninsured women in Almaty, Kazakhstan: a comparative cross-sectional study
Source: Front Glob Womens Health. 2025 Jul 25;6:1580888. doi: 10.3389/fgwh.2025.1580888 (PMC12331730; doi:10.3389/fgwh.2025.1580888)
Supplement: Supplementary file 1 [file Table1.docx]

Supplementary Table 1. Socio-demographic Characteristics of Insured and Uninsured Patients in a Hospital, Almaty Region, Kazakhstan (n = 107)

| **Variables** | **Categories** | **Insured (%)** | **Uninsured (%)** | **p-value** |
| --- | --- | --- | --- | --- |
| Age | <25 years | 38 (44,2%) | 14 (66,7%) | 0.116 |
|  | 25–34 years | 20 (23,3%) | 5 (23,8%) |  |
|  | 35–44 years | 16 (18,6%) | 0 (0,0%) |  |
|  | 45–54 years | 12 (14,0%) | 2 (9,5%) |  |
| Marital status | Married | 58 (67.4%) | 7 (33.3%) | 0.030* |
|  | Single | 13 (15.1%) | 7 (33.3%) |  |
|  | Divorced | 14 (16.3%) | 7 (33.3%) |  |
|  | Widowed | 1 (1.2%) | 0 (0.0%) |  |
| Educations | Secondary | 40 (46,5%) | 11 (52,4%) | 0.818 |
|  | Higher | 39 (45,3%) | 9 (42,9%) |  |
|  | Postgraduate | 7 (8,1%) | 1 (4,8%) |  |
| Residence | Urban | 59 (68,6%) | 27 (31,4%) | 0.513 |
|  | Rural | 15 (71,4%) | 6 (28,6%) |  |
| Income | <129,277 tenge | 62 (72,1%) | 24 (27,9%) | 0.063 |
|  | >129,277 tenge | 19 (90,5%) | 2 (9,5%) |  |
