# Supplementary material for: Exploring disparities in satisfaction with obstetric-gynecological care among insured and uninsured women in Almaty, Kazakhstan: a comparative cross-sectional study
Source: Front Glob Womens Health. 2025 Jul 25;6:1580888. doi: 10.3389/fgwh.2025.1580888 (PMC12331730; doi:10.3389/fgwh.2025.1580888)
Supplement: Supplementary file 2 [file Table2.docx]

**Supplementary Table 2. Comparative Analysis of Patient Satisfaction with Hospital Services Based on Insurance Status in the Almaty Region, Kazakhstan (n = 107)**

| **Variables** | **CBHI status** | **Categories** | | | |
| --- | --- | --- | --- | --- | --- |
|  |  | **Satisfied**  **N (%)** | **Somewhat Satisfied**  **N (%)** | **Somewhat Dissatisfied**  **N(%)** | **Dissatisfied**  **N(%)** |
| Perception of the Hospitalization Process | Uninsured | 20  (95,2%) | 0  (0,0%) | 0  (0,0%) | 1  (4,8%) |
|  | Insured | 78  (90,7%) | 1  (1,2%) | 3  (3,5%) | 3  (3,5%) |
| Perception of Hospitalization Duration | Uninsured | 16  (76,2%) | 0  (0,0%) | 0  (0,0%) | 5  (23,8%) |
|  | Insured | 54  (62,8%) | 2  (2,3%) | 2  (2,3%) | 28  (32,6%) |
| Perception of Involvement in Treatment and/or Delivery Decisions | Uninsured | 19  (90,5%) | 0  (0,0%) | 0  (0,0%) | 2  (9,5%) |
|  | Insured | 61  (70,9%) | 1  (1,2%) | 2  (2,3%) | 22  (25,6%) |
| Doctors' Attitude, Behavior, and Communication | Uninsured | 16  (76,2%) | 0  (0,0%) | 0  (0,0%) | 5  (23,8%) |
|  | Insured | 65  (75,6%) | 3  (3,5%) | 1  (1,2%) | 17  (19,8%) |
| Nurses' Attitude, Behavior, and Communication | Uninsured | 21  (100,0%) | 0  (0,0%) | 0  (0,0%) | 0  (0,0%) |
|  | Insured | 83  (96,5%) | 1  (1,2%) | 2  (2,3%) | 0  (0,0%) |
| Other Clinic Staff's Attitude, Behavior, and Communication | Uninsured | 21  (100,0%) | 0  (0,0%) | 0  (0,0%) | 0  (0,0%) |
|  | Insured | 82  (95,3%) | 4  (4,7%) | 0  (0,0%) | 0  (0,0%) |
| Waiting Time for Hospitalization or Assistance | Uninsured | 21  (100,0%) | 0  (0,0%) | 0  (0,0%) | 0  (0,0%) |
|  | Insured | 81  (94,2%) | 5  (5,8%) | 0  (0,0%) | 0  (0,0%) |
| Quality of Room Accommodation | Uninsured | 21  (100,0%) | 0  (0,0%) | 0  (0,0%) | 0  (0,0%) |
|  | Insured | 82  (95,3%) | 3  (3,5%) | 1  (1,2%) | 0  (0,0%) |
| Availability, Quality, and Cleanliness of Toilets | Uninsured | 21  (100,0%) | 0  (0,0%) | 0  (0,0%) | 0  (0,0%) |
|  | Insured | 82  (95,3%) | 2  (2,3%) | 2  (2,3%) | 0  (0,0%) |
| Availability of Hot Water, Ability to Take a Shower | Uninsured | 21  (100,0%) | 0  (0,0%) | 0  (0,0%) | 0  (0,0%) |
|  | Insured | 83  (96,5%) | 2  (2,3%) | 1  (1,2%) | 0  (0,0%) |
| Quality of Provided Food | Uninsured | 21  (100,0%) | 0  (0,0%) | 0  (0,0%) | 0  (0,0%) |
|  | Insured | 79  (91,9%) | 5  (5,8%) | 2  (2,3%) | 0  (0,0%) |
| Quality of Diagnostic Procedures | Uninsured | 20  (95,2%) | 1  (4,8%) | 0  (0,0%) | 0  (0,0%) |
|  | Insured | 82  (95,3%) | 3  (3,5%) | 1  (1,2%) | 0  (0,0%) |
| Quality of Procedures and Interventions | Uninsured | 21  (100,0%) | 0  (0,0%) | 0  (0,0%) | 0  (0,0%) |
|  | Insured | 82  (95,3%) | 3  (3,5%) | 3  (3,5%) | 0  (0,0%) |
| Medication Supply | Uninsured | 81  (94,2%) | 3  (3,5%) | 2  (2,3%) | 0  (0,0%) |
|  | Insured | 81  (94,2%) | 3  (3,5%) | 2  (2,3%) | 0  (0,0%) |
| Overall Medical Service | Uninsured | 21  (100,0%) | 21  (100,0%) | 21  (100,0%) | 0  (0,0%) |
|  | Insured | 82  (95,3%) | 4  (4,7%) | 0  (0,0%) | 0  (0,0%) |
