# Supplementary material for: Exploring disparities in satisfaction with obstetric-gynecological care among insured and uninsured women in Almaty, Kazakhstan: a comparative cross-sectional study
Source: Front Glob Womens Health. 2025 Jul 25;6:1580888. doi: 10.3389/fgwh.2025.1580888 (PMC12331730; doi:10.3389/fgwh.2025.1580888)
Supplement: Supplementary file 3 [file Table3.docx]

**Supplementary Table 3. Comparison of Hospitalization Methods Between Insured and Uninsured Patients: Distribution and Statistical Analysis**

| **Variables** | **Categories** | **Insured (%)** | **Uninsured (%)** | **P-value** |
| --- | --- | --- | --- | --- |
| Hospitalization Method | Emergency Care | 54 (79,4%) | 14 (20,6%) | 0,010 |
|  | Self-Referral | 24 (96,0%) | 1 (4,0%) |  |
|  | Referral by Doctor | 8 (57,1%) | 6 (42,9%) |  |
