# Supplementary material for: Exploring disparities in satisfaction with obstetric-gynecological care among insured and uninsured women in Almaty, Kazakhstan: a comparative cross-sectional study
Source: Front Glob Womens Health. 2025 Jul 25;6:1580888. doi: 10.3389/fgwh.2025.1580888 (PMC12331730; doi:10.3389/fgwh.2025.1580888)
Supplement: Supplementary file 4 [file Table4.docx]

**Supplementary Table 4. Binary and multivariable logistic regression analysis to identify factors associated with satisfaction of patients (n =107)**

| **Variables** | **Not satisfied** | **Satisfied** | **at binary level** | | **Multi-variable level** | |
| --- | --- | --- | --- | --- | --- | --- |
|  |  |  | **COR (95%CI)** | **p-value** | **AOR (95% CI)** | **p-value** |
| **Age** | 50 | 57 | 0.95 (0.90, 1.01) | 0.15 | 0.96 (0.91, 1.01) | 0.13 |
| **Gender** | 45 | 63 | 2.13 (0.98, 4.64) | 0.057 | 1.91 (0.88, 4.17) | 0.10 |
| **Region** | 55 | 52 | 0.45 (0.10, 2.17) | 0.32 | 0.36 (0.08, 1.71) | 0.20 |
| **Education** | 60 | 47 | 0.51 (0.16, 1.67) | 0.26 | 0.55 (0.17, 1.70) | 0.30 |
| **Income** | 40 | 67 | 0.39 (0.07, 2.24) | 0.29 | 0.33 (0.05, 1.85) | 0.21 |
| **Insurance Status** | 30 | 77 | 0.11 (0.02, 0.67) | 0.017 | 0.15 (0.03, 0.81) | 0.03 |
| **Kidney Disease** | 20 | 87 | 0.16 (0.03, 0.83) | 0.03 | 0.13 (0.03, 0.63) | 0.01 |
| **Vascular Disease** | 25 | 82 | 0.06 (0.002, 2.19) | 0.13 | 0.05 (0.002, 1.74) | 0.10 |
| **Days of Hospitalization** | 35 | 72 | 0.64 (0.42, 0.98) | 0.04 | 0.62 (0.41, 0.93) | 0.02 |
| **Complications (Main Dx)** | 15 | 92 | 1.82 (0.94, 3.48) | 0.07 | 1.77 (1.00, 3.34) | 0.05 |
